# Population validation of reproductive gene mutation loci and association with the litter size in Nubian goat

**DOI:** 10.5194/aab-64-375-2021

**Published:** 2021-09-17

**Authors:** Sanbao Zhang, Xiaotong Gao, Yuhang Jiang, Yujian Shen, Hongyue Xie, Peng Pan, Yanna Huang, Yingming Wei, Qinyang Jiang

**Affiliations:** 1 College of Animal Science and Technology, Guangxi University, Nanning 530004, Guangxi, China; 2 Institute for New Rural Development, Guangxi University, Nanning 530004, Guangxi, China

## Abstract

Litter size is an important component trait of doe reproduction. By improving it, production efficiency and economic benefits can be significantly provided. Genetic marker-assisted selection (MAS) based on proven molecular indicators could enhance the efficacy of goat selection, as well as litter size trait. Many molecular markers have been identified that they can be used to improve litter size in different goat breeds. However, the presence and value of these markers vary among goat breeds. In the present study, we used the reported loci on other breeds of goat as candidate loci to detect whether these loci appear in this Nubian goat population; then we proceed to genotype and detect surrounding loci (50 bp) by multiplex PCR and sequencing technology. As a result, 69 mutations (59 SNPs and 10 indels) were screened out from 23 candidate genes in Nubian goat population, 12 loci were significantly associated with the litter size of first-parity individuals; 5 loci were significantly associated with the litter size of second-parity individuals; 3 loci were significantly associated with the litter size of third-parity individuals. In addition, five loci were significantly associated with the average litter size. The additive effect value of *KITLG*: g.18047318 G>A in first parity, *KITLG*: g.18152042G>A in third parity, *KISS-1*: g.1341674 C>G in first parity, and *GHR*: g.32134187G>A in second parity exceed more than 0.40, and the preponderant alleles are G, C, A and G, respectively. Further, linkage disequilibrium analysis of 21 mutation loci shows that 3 haplotype blocks are formed, and the litter size of combination type AACC in *KISS-1* gene and AAGG in *KITLG* gene are significantly lower than that of other combinations genotype in first parity (P<0.05). These findings can provide effective candidate DNA markers for selecting superior individuals in Nubian goat breeding.

## 1 Introduction

The goat (*Capra hircus*) is believed to be one of the first domesticated ungulates that underwent domestication approximately 10 000 years ago; therefore it has been a witness to the historical progress of human civilization (Naderi et al., 2008). A variety of natural or artificial factors (e.g., environmental changes, human migration and socioeconomic influences) have shaped the phenotypic diversity of goat, which is one of the most important agricultural animals for various products (e.g., milk, meat and fiber) to humans (Silpa et al. 2018; Li et al. 2012; Takada et al. 1997b). The Nubian goat has good adaptability and reproductive performance with more than two kids per litter. They are farmed on a large scale in southwest China and also used to improve local doe.

Litter size is considered a trait with low heritability ($h^{2}=0.10-0.15$) (Liu et al., 2019), and traditional direct selection is ineffective in goat breeding (Cui et al., 2018). With the rapid development of animal molecular genetics and modern molecular biotechnology, the analysis of single gene or chromosome fragments has become the main object of analyzing fecundity traits. Marker-assisted selection (MAS), based on relevant genetic variants, is used with traits of low heritability ($h^{2}$), such as those associated with reproduction (Yh et al., 2020). The combined efficiency is significantly higher than that achievable by breeding based on the phenotype alone (Yuan et al., 2019). To facilitate MAS application to litter size in the goat industry, critical genetic variants causing phenotypic advantage should be verified, which is more convenient and efficient. Therefore, it is an effective way to scan single nucleotide polymorphisms (SNPs) in genes with known reproductive physiology functions. Multiplex PCR targeted amplification sequencing (MAT-seq) is an accurate, sensitive, widely applicable and economical method for loci detection and genotyping, which is a multiplex PCR amplification fragment compatible with Illumina HiSeq/MiSeq platform sequencing technology (Yoshihiko et al., 2018; Grandell et al., 2016). It is a good method to detect target sites or fragments (< 50 bp) and to genotype important candidate genes.

Based on current literature, some genes that affect the secretion of reproductive hormones in the hypothalamic–pituitary–testicular axis, ovulation rate, embryonic/fetal survival and endometrial receptivity are considered to be the main candidate genes affecting litter size (Xu et al., 2018). The influence of candidate genes on reproductive traits differs considerably because these genes could affect the physiological pathways, metabolism and phenotypic expression differentially with different paracrine or autocrine effects (Wang et al., 2018b). The major focus of goat breeding for genetic improvement greatly relies on candidate genes, which influence reproductive traits so as to ascertain the functions of genes as causing changes in and association with phenotypic values of the trait, molecular structure analysis, expression profile, sequence variability and their association with phenotypes. The selection for favorable alleles of candidate genes could help the development of high litter size goat breeding. Therefore, to provide an improved understanding of the genetic complexity underlying the litter size in Nubian goat, the objectives of the present study were the following: (1) to detect 43 previously reported mutations in the 24 genes associated with the litter size of other goat breeds (e.g., Shaanbei White Cashmere goat, Jining Grey goat, Boer goat) and whether they appear in this Nubian goat population; (2) to investigate the effects of loci involved in reproduction on litter size in Nubian goat; and (3) to explore the effects of locus–locus combinations on litter size. These information will be useful in identifying valuable genetic markers for MAS in Nubian goat.

**Figure 1:**
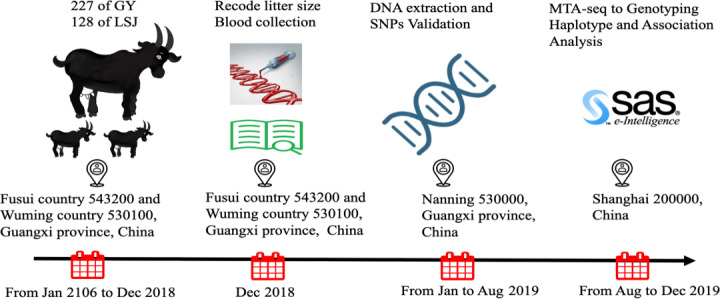
Schematic diagram of the experimental protocol across times. GY: Guangxi Fusui Guangyang Agricultural and Livestock Co. Ltd.; LSJ: Guangxi Lvshijie Agricultural investment co. Ltd.

## 2 Materials and methods

### 2.1 Animals and DNA extraction

The study was approved by the ethics committee of Guangxi University (Nanning, Guangxi, China) (approval number: 2016-Gxu205). A total of 355 Nubian doe (aged 12–36 months) were accessed from two farms (227 of Guangxi Fusui Guangyang Agricultural and Livestock Co. Ltd. and 128 of Guangxi Lvshijie Agricultural investment co. Ltd.) in Guangxi Province, China. All doe were mated naturally and raised under identical management conditions. Additionally, all selected individuals were healthy and with detailed reproduction records (litter size of first-, second- and third-parity individuals) from 2016 to 2018. The experimental protocol across times is shown in Fig. 1. All efforts were made to minimize any discomfort during blood collection. Approximately 5.0 mL of blood has been collected aseptically from the jugular vein of each doe into 5 mL vacutainer tubes containing the anticoagulant, ethylenediaminetetraacetic acid (EDTA-K2). Genomic DNA is extracted from blood using the DNeasy Blood and QIAamp DNA extraction kit (Qiagen, Germany). The quality of DNA is checked by a spectrophotometer taking ratio of optical density (OD) value at 260 and 280 nm. The integrity of DNA was detected by 1.0 % agarose gel electrophoresis. DNA samples were each diluted to a working concentration of 30 ng/µL by TE buffer (pH 8.0) and stored at -20 ${}^{\circ }$C for DNA pooling and genotyping.

**Table 1:** Genetics variants associated with the fecundity in goat.

Gene	Molecular marker	Founder breeds	Reference
*CSN1SI*	11 bp indel	Shaanbei White Cashmere goat	Wang et al. (2018a)
*CTNNB1*	26 bp indel	Shaanbei White Cashmere goat	Zhang et al. (2018)
*GHR*	14 bp duplicated deletion	Shaanbei White Cashmere goat	Yang et al. (2017)
*GDF9*	12 bp indel; Q320P and V397I; g.4093G>A	Shaanbei White Cashmere goat; Xinong Saanen dairy goat; Guanzhong dairy goat; Boer goat	Wang et al. (2017, 2019), An et al. (2013b)
*GNRH1*	g.3548A>G	Xinong Saanen dairy goat; Guanzhong dairy goat; Boer goat	An et al. (2013b)
*GNRHR*	g.-29T>G	West African Dwarf goat	Bemji et al. (2018)
*KDM6A*	16 bp and 5 bp indel	Shaanbei White Cashmere goat	Cui et al. (2018)
*KISS1*	g.2124T>A, g.2270C>T; T121A	Xinong Saanen dairy goat, Guanzhong dairy goat; Boer goat; Baladi goat, Zaraibi goat and Damascus goat	An et al. (2013c), El-Tarabany et al. (2017)
*KITLG*	g.13090G>T and g.13664C>A	Xinong Saanen dairy goat and Guanzhong dairy goat	An et al. (2015b)
*IGF1*	g.224A>G and g.227C>T	Malabari goat and Attappady Black goat	Thomas et al. (2016)
*NGF*	A705G	Xinong Saanen dairy goat; Guanzhong dairy goat	An et al. (2013a)
*PDGFRB*	10 bp indel	Shaanbei White Cashmere goat	Yang et al. (2019)
*POU1F1*	c.682G>T and c.837T>C	Shaanbei White Cashmere goat	Zhu et al. (2019)
*PRLR*	g.151435C>T and g.173057T>C; g.62130C>T	Shaanbei White Cashmere goat	An et al. (2015a); Hou et al. (2014)
*MARCH1*	7 bp, 15 bp, and 18 bp indels	Shaanbei White Cashmere goat	Kang et al. (2019)
*CYM*	Exon 8:c.T916C	Laoshan dairy goat	Lai et al. (2016)
*CDH26*	Exon8:c.T1063C; c.G1035A; c.T1034C	Laoshan dairy goat	Lai et al. (2016)
*NEDD4*	g.48710049	Laoshan dairy goat	Lai et al. (2016)
*INHA*	c.C126G; c.C936T	Xinong Saanen dairy goat, Guanzhong dairy goat and Boer goat	Hou et al. (2012)
*FOLR1*	g.7884A>C;	Boer goat	Hou et al. (2014)
*ATBF1*	g.25748G>A	Hainan Black goat	Zhang et al. (2015)
*SIRT3*	c.691C >T	Malabari and Attappady Black goat	Silpa et al. (2018)
*INH*βA	g.C936T	Xinong Saanen dairy goat, Guanzhong dairy goat and Boer goat	Hou et al. (2012)
*PGR*	g.74589762	Nubian goat	Preliminary work

### 2.2 Gene selection, primer design, DNA pool PCR amplification, polymorphism identification

We hypothesized that molecular markers identified on goat breeds (e.g., Shaanbei White Cashmere goat, Jining Grey goat, Boer goat) that are significantly associated with litter size would have the same effect on Nubian goat. In the present study, genes with known biological functions in follicular and ovarian development were first selected as target genes. These genes or SNPs identified in this study and known to influence reproductive traits in a goat breed were then selected as candidate genes or markers. Therefore, 43 candidate loci (29 SNPs and 14 indels) on 24 genes with known reproductive physiology functions in animals were selected as potential candidate loci for goat litter size (Table 1). Among the 43 candidate loci (Table S1), 38 polymorphic loci (29 SNPs and 9 indels) of 23 genes were verified by Sanger sequencing of PCR products amplified from 5 DNA pools. These DNA pools comprised 30 randomly selected individual genomic DNA samples as a polymerase chain reaction (PCR) template for amplification. Specific primers for PCR were designed based on these candidate gene sequences. The PCR was performed in a volume of 40 µL, containing 20 µL 2 × Taq PCR MasterMix, 1.5 µL each of forward and reverse primers, 2 µL pool DNA (30 ng/µL), and 15 µL double-distilled H${}_{2}$O. The PCR protocol was as follows: initial denaturation for 3 min at 95 ${}^{\circ }$C; denaturation for 15 s at 95 ${}^{\circ }$C; annealing for 30 s at 50–60 ${}^{\circ }$C; extension for 15–45 s at 72 ${}^{\circ }$C; and a final extension for 5 min at 72 ${}^{\circ }$C, with subsequent cooling to 4 ${}^{\circ }$C. The PCR products were detected by 2.0 % agarose gel electrophoresis. The purified PCR products were sequenced using Sanger sequencing, and the sequences were compared using DNASTAR and Chromas software to detect putative SNPs. The identified SNPs were individually genotyped by the MTA-seq method (Sangon Biotech, Shanghai, China). Three DNA samples were randomly selected from 355 samples to genotype twice, and 2 blank samples (double-distilled water) were also used to eliminate cross-contamination.

### 2.3 Multiplex PCR and sequencing to genotype and detect surrounding loci (50 bp)

A panel which contains 38 target SNPs loci was designed (Table S2). Library preparation is performed by two-step PCR. First round PCR reaction was set up as follows: DNA (10 ng/µL) 2 µL; amplicon PCR forward primer mix (10 µM) 1 µL; amplicon PCR reverse primer mix (10 µM) 1 µL; 2 × PCR Ready Mix 15 µL (total 25 µL) (Kapa HiFi Ready Mix). The plate was sealed, and PCR was performed in a thermal instrument (BIO-RAD, T100TM) using the following program: 1 cycle of denaturing at 98 ${}^{\circ }$C for 5 min, first 8 cycles of denaturing at 98 ${}^{\circ }$C for 30 s, annealing at 50 ${}^{\circ }$C for 30 s, elongation at 72 ${}^{\circ }$C for 30 s, then 25 cycles of denaturing at 98 ${}^{\circ }$C for 30 s, annealing at 66 ${}^{\circ }$C for 30 s, elongation at 72 ${}^{\circ }$C for 30 s, extension at 72 ${}^{\circ }$C for 5 min and finally held at 4 ${}^{\circ }$C. The PCR products were checked by using electrophoresis in 1 % (w/v) agarose gels in TBE buffer (Tris, boric acid, EDTA) stained with ethidium bromide (EB) and visualized under UV light. Then we used AMPure XP beads to purify the amplicon product. After that, the second-round PCR was performed. PCR reaction was set up as follows: DNA (10 ng/µL) 2 µL; universal P7 primer with barcode (10 µM) 1 µL; universal P5 primer (10 µM) 1 µL; 2 × PCR Ready Mix 15 µL (total 30 µL) (Kapa HiFi Ready Mix). The plate was sealed and PCR was performed in a thermal instrument (BIO-RAD, T100TM) by using the following program: 1 cycle of denaturing at 95 ${}^{\circ }$C for 3 min, then 5 cycles of denaturing at 94 ${}^{\circ }$C for 30 s, annealing at 55 ${}^{\circ }$C for 20 s, elongation at 72 ${}^{\circ }$C for 30 s, elongation at 72 ${}^{\circ }$C for 30 s and a final extension at 7 ${}^{\circ }$C for 5 min. Then we used AMPure XP beads to purify the amplicon product. The libraries were then quantified and pooled. Paired-end sequencing of the library is performed on the HiSeqXTen sequencers (Illumina, San Diego, CA).

### 2.4 Data quality control (QC) and SNP calling

Raw reads were filtered according to two steps: removing low-quality bases from reads 3${}^{\prime }$ to 5${}^{\prime }$ (Q<20) by PRINSEQ-lite (v 0.20.3), and the remaining clean data were mapped to the reference genome by BWA (version 0.7.13-r1126) with default parameters. A Perl script was written to calculate each genotype of target locus. ANNOVAR (16 April 2018) was used to detect genetic variants.

### 2.5 Association analysis

Statistical analyses were performed using SAS (V. 9.2) (SAS Institute Inc.). The scattergrams of litter size at different parity were computed using Microsoft Excel (v16.42). The general linear model (GLM) of the analysis of variance (ANOVA) was used to analyze the relationships between the different genotypes and litter size. Because these two goat farms are national core farms, the geographical location is very close and all goats were fed with standardized feeding method. The effect of the farm on the number of goats' litter size is very small, which can be ignored. And least-squares mean (LSM) was used for multiple comparisons in litter size of goat with different loci genotypes according to the adjusted linear model:

1 $$Y_{ijk}=\mu +G_{i}(+P_{j})+W_{k}+\varepsilon_{ijk}.$$

In this model, $Y_{ijk}$ represents phenotypic value (litter size); μ is the population mean; $P_{j}$ shows the effect of the $j^{\text{th}}$ parity (j= first, second, third or average); $G_{i}$ represents the effect of the $i^{\text{th}}$ genotypes (i = Ref/Ref, Ref/Alt, or Alt/Alt); $W_{k}$ represents the effect of the $k^{\text{th}}$ weather (k = cool or hot) and $\varepsilon_{ijk}$ represents random error. P values < 0.05 were considered to be significant. All statistically significant single marker traits associated with LSM litter size were initially included in further combined haplotypes analysis of the combined effects of different genes on LSM litter size.

To estimate the mode of inheritance of the loci, the following functions were used:

2 $$\begin{array}{rl} & D=A_{1}A_{0}-1/2(A_{1}A_{1}+A_{0}A_{0})\\ & A=1/2(A_{1}A_{1}-A_{0}A_{0}) \end{array},$$

where D and A are the estimated values of dominant effect and additive effect, respectively; $A_{1}A_{1}$, $A_{1}A_{0}$ and $A_{0}A_{0}$ are the genotypic values of dominant homozygote, heterozygote and allozygote, respectively. The genetic effects are partially recessive, incompletely dominant, partially dominant, complete dominant and over dominant when D<0, D=0, 0<D<|A|, D=|A| and D>|A|, respectively (Chong et al., 2018).

The substitution effect of alleles was calculated by linear regression model SAS (V. 9.2):

3 $$Y_{ij}=\mu +bX_{ij}+\varepsilon_{ij}.$$

In this model, $Y_{ij}$ represents phenotypic value (litter size); μ is the population mean; b is the corresponding regression coefficient; X ${}_{ij}$ is the value of the proportion of each allele in the genotype and $\varepsilon_{ij}$ represents random error.

### 2.6 Population parameter calculation

According to the results of genotype statistical analysis, genotypic and allelic frequencies, polymorphism information content (PIC), genetic homozygosity (Ho), genetic heterozygosity (He), effective allele number (Ne) and the Hardy–Weinberg equilibrium (HWE) were analyzed by POPGENE software version 3.2. Distribution differences for loci genotypic frequencies in Nubian goats were performed using the chi-square test in SPSS (Version 18.0) (IBM Corp., Armonk, NY, USA). Nubian goat populations with P>0.05 (chi-square test) were considered to conform to the Hardy–Weinberg equilibrium.

### 2.7 Haplotype analysis

In the current study, only the significant SNPs in the same gene were used to estimate the extent of linkage disequilibrium (LD) and haplotype blocks. The extent of LD between significant SNP pairs and haplotype blocks was estimated using the HaploView 4.2 program. Using the default setting of HaploView 4.2 to run the program, the parameter $r^{2}$ represents the statistical association between the two SNPs. Generally, when $r^{2}>0.8$, the two SNPs can be regarded as closely linked and can be replaced each other. The association analysis between haplotype region and litter size, Hap score and p value of Hap score were computed using R software (haplo.states packages). The model of association analysis between combined haplotypes and litter size is as follows in Eq. (1) except that the genotype (G) is replaced by combined haplotypes.

## 3 Results

### 3.1 Litter size trait analysis

**Figure 2:**
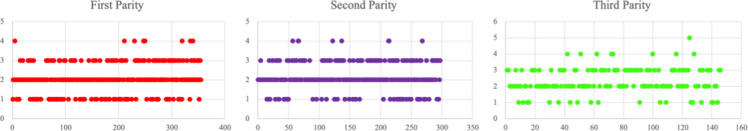
Scatterplots of litter size for the three parities. A dot represents a Nubian goat.

Litter size from three parities was recorded in this study. The average litter size at the first to third parity was 2.130 ± 0.022; the litter size at the first parity was 2.065 ± 0.037; the second-parity litter size was 2.168 ± 0.040; and the third-parity litter size was 2.343 ± 0.066. Scatterplots also show that litter size tended to increase with the parity (Fig. 2).

### 3.2 Verification of polymorphic loci, genotyping and quality control

Among the 43 candidate loci (29 SNPs and 14 indels) of 24 genes, 38 polymorphic loci (29 SNPs and 9 indels) of 23 genes were identified by DNA pooling and Sanger sequencing (Table S2). Then, the primers of multiplex PCR were redesigned and sequenced. The genotypic correlation coefficients of three pairs of technical repeats equal to one and no genotyping signals were detected in two blank samples, indicating that the MTA-seq method is reliable. Sixty-nine mutations (59 SNPs and 10 indels) were screened out from 23 candidate genes by multiplex PCR and sequencing technology (Date S3).

### 3.3 Single loci polymorphisms associated with litter size

Twenty-one mutations (19 SNPs and 2 indels) were significantly linked with litter size of Nubian goat population. Twelve loci were significantly associated with the litter size of first-parity individuals, including *POU1F1*: g.34236170A>G, *KITLG*: g.18047318G>A and g.18152042G>A, *MARCHF1*: g.1885620A>G, *GDF9*: g.66027701C>T, *NEDD4*: g.48710049G>A, *PGR*: g.74589762C>T, *KISS-1*: g.1341600A>G and g.1341674C>G, *GHR*: g.32134266T>C, *CTNNB1*: c.1684-238_1684-222delACTTGGCTGTGCACAGT and c.1684-218_1684-211delGGAGGTGCinsT; five loci (*KITLG*: g.18048657G>T, *MARCHF1*: g.1858739G>A, *NEDD4*: g.48709794G>A, *PGR*: g.74589762C>T, *GHR*: g.32134187G>A) were significantly associated with the litter size of second-parity individuals; three loci (*KITLG*: g.18152042G>A, *ATBF1*: g.39535967C>T and g:39535996C>T) were significantly associated with the litter size of third-parity individuals. In addition, five loci were significantly associated with the average litter size, including *INHA*: g.2838305G>ins-C, g:28318345C>G and g:28318349G>C, *KITLG*: g.18047318G>A, *GHR*: g.32134266T>C. The additive effect values of *KITLG*: g.18047318 G>A in first parity and *KITLG*: g.18152042G>A in third parity, *KISS-1*: g.1341674 C>G in first parity, *GHR*: g.32134187G>A in second parity exceed more than 0.40, and the preponderant alleles are G, C, A and G, respectively. These significantly correlated loci detailed information is provided in Table 2. Other loci not significantly associated with litter size in any parity were excluded for further analysis.

**Table 2:** Significantly association loci between the 21 polymorphism loci (19 SNPs and 2 indels) and litter size in Nubian goat.

Birth order	Gene	Chromosome	Start	End	Ref	Alt	No. of does			Litter size mean ± SE			P	Effects		
							Ref/ Ref	Ref/ Alt	Alt/ Alt	Ref/Ref	Ref/Alt	Alt/Alt	value	Additive effect	Dominant effect	Substitute effect
First parity	*POU1F1*	NC_030808.1	34236170	34236170	A	G	182	141	32	2.259 ± 0.154${}^{\text{b}}$	2.163 ± 0.152${}^{\text{b}}$	2.410 ± 0.191${}^{\text{a}}$	0.043	0.075 ± 0.067	-0.172 ± 0.086	0.170 ± 0.099
	*KITLG*	NC_030812.1	18047318	18047318	G	A	267	83	5	2.214 ± 0.147${}^{\text{a}}$	2.220 ± 0.165${}^{\text{a}}$	1.355 ± 0.334${}^{\text{b}}$	0.013	-0.429 ± 0.150	0.436 ± 0.167	-0.188 ± 0.085
		NC_030812.1	18152042	18152042	G	A	244	105	6	2.181 ± 0.153${}^{\text{b}}$	2.234 ± 0.153${}^{\text{b}}$	2.645 ± 0.314${}^{\text{a}}$	0.038	0.232 ± 0.141	-0.179 ± 0.156	0.133 ± 0.080
	*MARCHF1*	NC_030813.1	1885620	1885620	A	G	291	59	5	2.198 ± 0.147${}^{\text{a}}$	2.451 ± 0.170${}^{\text{a}}$	1.897 ± 0.334${}^{\text{b}}$	0.014	-0.151 ± 0.153	0.404 ± 0.176	-0.374 ± 0.241
	*GDF9*	NC_030814.1	66027701	66027701	C	T	185	154	16	2.123 ± 0.152${}^{\text{b}}$	2.268 ± 0.150${}^{\text{b}}$	2.662 ± 0.223${}^{\text{a}}$	0.004	0.269 ± 0.088	0.124 ± 0.102	0.338 ± 0.134
	*NEDD4*	NC_030817.1	48710049	48710049	G	A	297	56	2	2.241 ± 0.147${}^{\text{b}}$	1.962 ± 0.170${}^{\text{b}}$	2.764 ± 0.510${}^{\text{a}}$	0.009	0.262 ± 0.248	-0.541 ± 0.263	0.037 ± 0.125
	*PGR*	NC_030822.1	74589762	74589762	C	T	239	104	12	2.117 ± 0.157${}^{\text{b}}$	2.058 ± 0.166${}^{\text{b}}$	2.574 ± 0.217${}^{\text{a}}$	0.022	0.229 ± 0.105	-0.287 ± 0.123	0.388 ± 0.163
	*KISS-1*	NC_030823.1	1341600	1341600	A	G	43	166	146	1.957 ± 0.174${}^{\text{b}}$	2.258 ± 0.154${}^{\text{a}}$	2.266 ± 0.152${}^{\text{a}}$	0.016	0.154 ± 0.059	0.147 ± 0.079	0.073 ± 0.057
		NC_030823.1	1341674	1341674	C	G	293	60	2	2.195 ± 0.148${}^{\text{a}}$	2.348 ± 0.168${}^{\text{a}}$	1.340 ± 0.498${}^{\text{b}}$	0.020	-0.428 ± 0.239	0.581 ± 0.254	-0.749 ± 0.373
	*GHR*	NC_030827.1	32134266	32134266	T	C	304	47	4	2.249 ± 0.148${}^{\text{b}}$	1.987 ± 0.172${}^{\text{b}}$	2.744 ± 0.367${}^{\text{a}}$	0.014	0.247 ± 0.171	-0.509 ± 0.195	0.034 ± 0.100
	*CTNNB1*	NC_030829.1	13712297	13712314	TACTTGGCTGTGCACAGT	del_TACTTGGCTGTGCACAGT	349	6	–	2.225 ± 0.148${}^{\text{a}}$	1.697 ± 0.308${}^{\text{b}}$		0.036	–	–	–
		NC_030829.1	13712318	13712325	GGAGGTGC	del_GGAGGTGC	349	6	–	2.225 ± 0.148${}^{\text{a}}$	1.697 ± 0.308${}^{\text{b}}$	–	0.036	–	–	–
Second parity	*KITLG*	NC_030812.1	18048657	18048657	G	T	135	136	26	2.193 ± 0.088${}^{\text{a}}$	2.249 ± 0.087${}^{\text{a}}$	1.848 ± 0.141${}^{\text{b}}$	0.032	-0.172 ± 0.076	0.228 ± 0.096	-0.299 ± 0.114
	*MARCHF1*	NC_030813.1	1858739	1858739	G	A	290	7	–	2.153 ± 0.073${}^{\text{b}}$	2.682 ± 0.274${}^{\text{a}}$	–	0.035	–	–	–
	*NEDD4*	NC_030817.1	48709794	48709794	G	A	102	145	50	2.172 ± 0.095${}^{ab}$	2.083 ± 0.082${}^{\text{b}}$	2.347 ± 0.115${}^{\text{a}}$	0.038	0.087 ± 0.061	-0.177 ± 0.085	0.010 ± 0.067
	*PGR*	NC_030822.1	74589762	74589762	C	T	205	82	10	2.206 ± 0.076${}^{\text{a}}$	2.080 ± 0.102${}^{\text{a}}$	1.717 ± 0.232${}^{\text{b}}$	0.038	-0.244 ± 0.116	-0.119 ± 0.138	-0.310 ± 0.180
	*GHR*	NC_030827.1	32134187	32134187	G	A	248	47	2	2.150 ± 0.075${}^{\text{b}}$	2.101 ± 0.116${}^{\text{b}}$	3.507 ± 0.497${}^{\text{a}}$	0.020	0.678 ± 0.249	-0.727 ± 0.267	2.276 ± 0.130
Third parity	*KITLG*	NC_030812.1	18152042	18152042	G	A	98	46	2	2.329 ± 0.134${}^{\text{a}}$	2.035 ± 0.155${}^{\text{a}}$	1.439 ± 0.578${}^{\text{b}}$	0.037	-0.445 ± 0.294	0.151 ± 0.319	-0.361 ± 0.158
	*ATBF1*	NC_030825.1	39535967	39535967	C	T	102	44	–	2.124 ± 0.128${}^{\text{b}}$	2.405 ± 0.163${}^{\text{a}}$	–	0.044	–	–	–
		NC_030825.1	39535996	39535996	C	T	102	44	–	2.124 ± 0.128${}^{\text{b}}$	2.405 ± 0.163${}^{\text{a}}$	–	0.044	–	–	–
Average	*INHA*	NC_030809.1	28318305	28318305	G	ins_C	348	450	–	2.380 ± 0.161${}^{\text{a}}$	2.271 ± 0.161${}^{\text{b}}$	–	0.004	–	–	–
		NC_030809.1	28318345	28318345	C	G	453	345	–	2.273 ± 0.161${}^{\text{b}}$	2.380 ± 0.161${}^{\text{a}}$	–	0.005	–	–	–
		NC_030809.1	28318349	28318349	G	C	348	366	84	2.379 ± 0.161${}^{\text{b}}$	2.259 ± 0.162${}^{\text{a}}$	2.317 ± 0.175${}^{\text{b}}$	0.015	-0.031 ± 0.044	-0.088 ± 0.057	-0.080 ± 0.041
	*KITLG*	NC_030812.1	18047318	18047318	G	A	597	191	10	2.326 ± 0.159${}^{\text{a}}$	2.342 ± 0.166${}^{\text{a}}$	1.744 ± 0.274${}^{\text{b}}$	0.011	–0.291 ± 0.113	0.308 ± 0.124	–0.121 ± 0.062
	*GHR*	NC_030827.1	32134266	32134266	T	C	694	98	6	2.339 ± 0.159${}^{\text{b}}$	2.185 ± 0.172${}^{\text{b}}$	2.909 ± 0.329${}^{\text{a}}$	0.016	0.285 ± 0.146	-0.440 ± 0.162	0.041 ± 0.080

Litter size is presented as the least squares mean ± standard error (SE) of phenotypic values. Different superscript letters indicate significant differences between groups (P<0.05). “–” denotes information is not available.

### 3.4 Population genetic parameters

The genotypes and allele frequencies, He, Ho, Ne, PIC and Hardy–Weinberg equilibrium test P values, as well as other genetic parameters, associated with the significantly correlated loci were calculated to determine the genotype distribution among Nubian goat (Table 3). The significantly correlated loci data indicated the minimum (maximum) values of He were 0.02 (0.46), the minimum (maximum) value of Ho was 0.52 (0.98) and the minimum (maximum) value of Ne was 1.02 (1.94). Thirteen loci were low polymorphic status (PIC < 0.25); other SNPs were moderate (0.25 < PIC < 0.50) or high polymorphic (PIC > 0.50) status. Five loci deviated from Hardy–Weinberg equilibrium (P<0.05).

**Table 3:** Population genetic parameters of the significantly associations loci in Nubian goat.

Gene	Chromosome	Start	End	Ref	Alt	No. of does				Allelic frequencies		Genotypic frequencies			Population parameters			PIC	$\chi^{2}$	P value
						Total	Ref/Ref	Ref/Alt	Alt/Alt	Ref	Alt	Ref/Ref	Ref/Alt	Alt/Alt	H0	HE	Ne		Chi square	
*POU1F1*	NC_030808.1	34236170	34236170	A	G	355	182	141	32	A	G	AA	AG	GG	0.59	0.41	1.70	0.33	0.39	0.82
										0.71	0.29	0.51	0.40	0.09						
*INHA*	NC_030809.1	28318305	28318305	G	ins_C	355	152	203	–	G	ins_C(0.29)	GG	G/ins_C	–	0.59	0.41	1.69	0.33	56.91	0.00
										0.71		0.43	0.57							
		28318345	28318345	C	G	355	204	151	–	C	G	CC	CG	–	0.67	0.33	1.50	0.33	25.90	0.00
										0.79	0.21	0.57	0.43							
		28318349	28318349	G	C	355	152	162	41	G	C	GG	GC	CC	0.55	0.45	1.82	0.35	0.05	0.98
										0.66	0.34	0.43	0.46	0.12						
*KITLG*	NC_030812.1	18047318	18047318	G	A	355	267	83	5	G	A	GG	GA	AA	0.77	0.23	1.29	0.20	0.26	0.88
										0.87	0.13	0.75	0.23	0.01						
		18048657	18048657	G	T	355	156	163	36	G	T	GG	GT	TT	0.56	0.44	1.79	0.34	0.48	0.79
										0.67	0.33	0.44	0.46	0.10						
		18152042	18152042	G	A	355	244	105	6	G	A	GG	GA	AA	0.72	0.28	1.38	0.24	1.97	0.37
										0.84	0.16	0.69	0.30	0.02						
*MARCHF1*	NC_030813.1	1858739	1858739	G	A	355	347	8	–	G	A	GG	GA	–	0.98	0.02	1.02	0.02	0.05	0.83
										0.99	0.01	0.98	0.02							
		1885620	1885620	A	G	355	291	59	5	A	G	AA	AG	GG	0.82	0.18	1.21	0.16	0.99	0.61
										0.90	0.10	0.82	0.17	0.01						
*GDF9*	NC_030814.1	66027701	66027701	C	T	355	185	154	16	C	T	CC	CT	TT	0.61	0.39	1.63	0.31	5.27	0.07
										0.74	0.26	0.52	0.43	0.05						
*NEDD4*	NC_030817.1	48709794	48709794	G	A	355	125	170	60	G	A	GG	GA	AA	0.52	0.48	1.94	0.37	0.03	0.99
										0.59	0.41	0.35	0.48	0.17						
		48710049	48710049	G	A	355	297	56	2	G	A	GG	GA	AA 0.01	0.85	0.15	1.18	0.14	0.13	0.93
										0.92	0.08	0.84	0.16							
*PGR*	NC_030822.1	74589762	74589762	C	T	355	239	104	12	C	T	CC	CT	TT	0.70	0.30	1.42	0.25	0.03	0.99
										0.82	0.18	0.67	0.29	0.03						
*KISS-1*	NC_030823.1	1341600	1341600	A	G	355	43	166	146	A	G	AA	AG	GG	0.54	0.46	1.84	0.35	0.16	0.92
										0.35	0.65	0.12	0.47	0.41						
		1341674	1341674	C	G	355	293	2	60	C	G	CC	CG	GG	0.72	0.28	1.40	0.24	341.08	0.00
										0.83	0.17	0.83	0.01	0.17						
*ATBF1*	NC_030825.1	39535967	39535967	C	T	355	251	104	–	C	T	CC	CT	–	0.75	0.25	1.33	0.22	10.46	0.00
										0.85	0.15	0.71	0.29							
		39535996	39535996	C	T	355	251	104	–	C	T	CC	CT	–	0.75	0.25	1.33	0.22	10.46	0.00
										0.85	0.15	0.71	0.29							
*GHR*	NC_030827.1	32134187	32134187	G	A	355	296	57	2	G	A	GG	GA	AA	0.84	0.16	1.19	0.14	0.18	0.92
										0.91	0.09	0.83	0.16	0.01						
		32134266	32134266	T	C	355	304	47	4	T	C	TT	TC	CC	0.86	0.14	1.17	0.13	1.93	0.38
										0.92	0.08	0.86	0.13	0.01						
*CTNNB1*	NC_030829.1	13712297	13712314	TACTTGGCTGTGCACAGT	del_TACTTGGCTGTGCACAGT	355	349	6	–	TACTTGGCTGTGCACAGT	del_TACTTGGCTGTGCACAGT	TT	TACTTGGCTGTGCACAGT/del_TACTTGGCTGTGCACAGT	–	0.98	0.02	1.02	0.02	0.03	0.87
										0.99	0.01	0.98	0.02							
		13712318	13712325	GGAGGTGC	del_GGAGGTGC	355	349	6	–	GGAGGTGC	del_GGAGGTGC	GG	GGAGGTGC/del_GGAGGTGC	–	0.98	0.02	1.02	0.02	0.03	0.87

A, G, C and T of the gene frequency and genotype frequency column respectively represent the adenine, guanine, cytosine and thymine; PIC, polymorphic information content; Ho, homozygosity; He, heterozygosity; Ne, number of effective allele; $\chi^{2}$ and p value denote the chi-square value and P value of Hardy–Weinberg equilibrium test; “–” denotes information is not available. $\chi^{2}0.05=5.991$, $\chi^{2}0.01=9.21$, df = 1. P<0.05 means the difference is significant, and P>0.05 means the difference is not significant.

**Figure 3:**
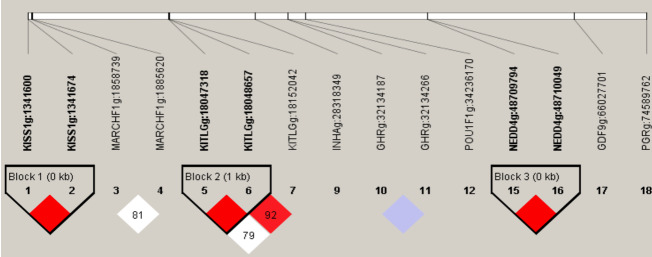
Linkage disequilibrium plot of 15 significant association loci. Intra-gene haplotypes (with blocks) are illustrated based on $r^{2}$ measurements. The numbers in blocks represent $r^{2}$ values. The degree of LD is reflected by the color intensity of the block. Red boxes are tag mutations included in association analysis. Linkage disequilibrium pattern for the significant SNPs in *KISS-1*, *KITLG* and *NEDD4 *gene. Scale of red color indicates the extend linkage disequilibrium ($D^{\prime }$ value).

### 3.5 Haplotype analysis

The $D^{\prime }$($r^{2}$) value is a factional indicator of LD and is calculated in this study. Three haplotype blocks were identified in Nubian goat. The $D^{\prime }$ ($r^{2}$) values are 1.00 (0.18), 1.00(0.075), 1.00(0.134) in Block 1 (*KISS-1*: g.1341600 A>G and *KISS-1*: g.1341674 C>G), Block 2 (*KITLG*: g.18047318 G>A and *KITLG*: g.18048657 G>T) and Block 3 (*KITLG*: g.18048657 G>T and *KITLG*: g.18152042 G>A), which are closely linked (Fig. 3), respectively. Block 1 has three different haplotypes: GC (64.5 %), AG (9.0 %) and AC (26.5 %). Block 2 has three different haplotypes: GT (33.1 %), GG (53.8 %) and AG (13.1 %). Block 3 has three different haplotypes: GG (59.2 %), AA (8.5 %) and AG (32.4 %) (Table 4).

**Table 4:** Haplotype association analysis of three blocks with litter size.

Blocks	Haplotypes	Hap frequency	Hap score	P value
Block 1	AC	0.26479	-2.57987	0.00988
	AG	0.09014	0.74552	0.45596
	GC	0.64507	1.99684	0.04584
Block 2	GG	0.53803	-1.05948	0.28938
	AG	0.13099	-0.98776	0.32327
	GT	0.33099	1.86348	0.06239
Block 3	AA	0.08451	-1.56236	0.1182
	GG	0.59155	-0.02334	0.98138
	AG	0.32394	0.92218	0.35643

Hap frequency: the haplotype frequency in the population; Hap score: statistical measurement of correlation between each haplotype and traits. The positive or negative value of the score indicates the frequency of the haplotype in the population with positive or negative effects on the litter size. P value: the progressive chi-square P value is calculated by the square of score statistics.

### 3.6 Association analysis of multiple markers with reproductive traits

According to the results of linkage disequilibrium, we combined the loci with linkage disequilibrium and analyzed the correlation with litter size (Table 5). Two combinations (Block 1 and Block 2) were founded to be significantly associated with litter size in the first parity (P<0.05). The litter size of combination type AACC in *KISS-1* gene and combination type AAGG in *KITLG *gene were obviously lower than that of other combinations.

## 4 Discussion

MAS has been widely used in the selection of important economic characters of pigs, chickens, cattle and sheep. In the same way, there has been some research progress in goat. But the molecular markers found in goat are various in different breeds. Considering research progress of goat molecular markers, being significantly associated with litter size, we hypothesized that molecular markers identified on goat breeds (e.g., Shaanbei White Cashmere goat, Jining Grey goat, Boer goat) would have the same effect on Nubian goat. Therefore, we verify the polymorphisms in Nubian goat population of reported loci significantly associated with litter size and then perform genotyping and detection of surrounding loci (50 bp). By doing so, we are able to provide some scientific data to enable the efficient and rapid development of the Nubian goat industry.

In this paper, 69 mutations were screened out from 23 candidate genes in Nubian goat by multiplex PCR and sequencing technology. However, only 21 mutations were significantly linked with litter size of Nubian goat. Interestingly, we found seven loci without mutation homozygous (*INHA*: g.2838305G>ins-C and g:28318345C>G; *MARCHF1*: g.1858739G>A; *ATBF1*: g.39535967C>T and g:39535996C>T; *CTNNB1*: c.1684-238_1684-222delACTTGGCTGTGCACAGT and c.1684-218_1684-211delGGAGGTGCinsT). It may be caused by the fact that homozygous lethal mutation and/or mutant homozygote is sterile (Nicol et al., 2009). In this sampling group of Nubian goat, 13 loci are of low polymorphic status (PIC < 0.25), which suggests that they have low selection potential; 8 loci are of moderate (0.25 < PIC < 0.50) or high polymorphic (PIC > 0.50) status, which suggests that they could provide more effective genetic information. Five loci obviously deviate from Hardy–Weinberg equilibrium (p<0.05) in Nubian goat, which is mainly due to artificial selection and/or nonrandom mating/or genetic drift (Wang et al., 2015).

**Table 5:** Significantly associations between the multiple markers and litter size in Nubian goat.

Birth	Haplotype	Genotypes	No. of	litter size	P value	Effects		
order			does	means ± SE		Additive effect	Dominant effect	Substitute effect
First parity	Block 1	AACC	27	1.867 ± 0.191${}^{\text{b}}$	0.0048	0.267 ± 0.244	0.610 ± 0.303	0.605 ± 0.381
		AACG	14	2.210 ± 0.225${}^{\text{a}}$				
		AGCC	120	2.203 ± 0.156${}^{\text{a}}$				
		AGCG	46	2.398 ± 0.175${}^{\text{a}}$				
		GGCC	146	2.264 ± 0.151${}^{\text{a}}$				
	Block 2	AAGG	5	1.333 ± 0.334${}^{\text{b}}$	0.0256	-0.379 ± 0.158	0.579 ± 0.187	-0.059 ± 0.122
		GAGG	47	2.292 ± 0.176${}^{\text{a}}$				
		GAGT	36	2.092 ± 0.187${}^{\text{a}}$				
		GGGG	104	2.130 ± 0.159${}^{\text{a}}$				
		GGGT	127	2.245 ± 0.151${}^{\text{a}}$				
		GGTT	36	2.244 ± 0.181${}^{\text{a}}$				

Litter size is presented as the least squares mean ± standard error (SE) of phenotypic values. Different superscript letters indicate significant differences between groups (P<0.05).

In these 21 polymorphic loci, they are also significantly associated with litter size of other goat population. For example, *KITLG*: g.18047318 G>A is revealingly associated with litter size of Xinong Saanen (SN), Guanzhong (GZ) and Boer goat (An et al., 2012); *GDF9*: g.66027701C>T is tellingly associated with litter size of Inner Mongolia cashmere goat (Nicol et al., 2009) as well. In quantitative genetics, the genetic variations of major economic traits are explained by both additive effect and dominant effect. Additive effect is considered to be a fixed component in the breeding, which also known as breeding value. Dominant effect refers to the deviation between the effect value of each gene and its additive effect value, which comes from the effect produced by the interaction between alleles. This effect is the main part of heterosis that can be inherited but not fixed in the breeding. The genetic effect value analysis of 21 significant loci showed that the additive effect values of *KITLG* as g.18047318 G>A in first parity, *KITLG* as g.18152042G>A in third parity, *KISS-1* as g.1341674 C>G in first parity, and *GHR* as g.32134187G>A in second parity exceed more than 0.40, and the preponderant alleles are G, C, A and G, respectively.

In haplotype structure analysis, three linkage disequilibrium blocks are found in these 21 loci. The haplotypes are integrated into a GLM model to explore the effect of loci–loci combinations on litter size. The litter size of combination type AACC in *KISS-1* gene and AAGG in *KITLG* gene are expressively lower than that of other combinations genotype in first born (P<0.05). Our findings indicated that homozygous individuals with the *KISS-1* gene AACC and *KITLG* gene AAGG genotype should not be retained in MAS breeding programs for improving the frequency of favorable alleles in the Nubian goat.

In the functional annotation of mutation loci (Table S4), there are five SNPs (*POU1F1*: g.34236170A>G; *GDF9*: g.66027701C>T; *NEDD4*: g.48710049G>A; *PGR*: g.74589762C>T; *KISS-1*: g.1341600 A>G) in the coding region (exon) which are meaningfully related to litter size, and three (*POU1F1*, *NEDD4*, *PGR*) of them are synonymous mutations. They may change the efficiency, stability and accuracy of mRNA alternative splicing (Kimchisarfaty et al., 2007; Duan et al., 2003) as well as protein translation and fold (Sauna and Kimchisarfaty, 2011) to affect litter size in doe. Another two (*KISS-1* and *GDF9*) directly change amino acids (*KISS-1*: p.Ala11Thr; *GDF9*: p.Ala273Val) and could change protein function, which maybe affects the secretion of reproductive hormone (e.g., *GH*, *PRL*, *TSH* β, *PGR*) or/and early folliculogenesis (Hanrahan et al., 2004; Silva et al., 2011) to affect litter size in doe. There are 16 loci located in the non-coding region (5_prime_UTR, 3_prime_UTR, intron). Three loci (*INHA*: g.2838305G>ins-C, g:28318345C>G and g:28318349G>C) are located in 5_prime_UTR. It may lead to the change of *INHA* gene frame shift and/or translation activity to affect estrus start and litter size in doe (Zi et al., 2012). Two loci are located in 3_prime_UTR; it may affect the binding sites of miRNA and the stability of mRNA, subcellular localization and translation level (Stark et al., 2005), which affect litter size in doe. Eleven loci are located in intron, which mainly involve in expression regulation and selective shearing. They may affect the cutting of precursor mRNA, cause exon skipping or intron inclusion, and lead to the change of protein sequence, which causes functional difference (Wang et al., 2014) to affect litter size in doe. This suggests that these mutations have potential functional significance, although the underlying mechanism remains to be elucidated in Nubian goat.

## 5 Conclusions

In conclusion, 38 previously reported mutations of 23 reproduction genes were identified in Nubian goat, which are importantly related to litter size of other breeds. A total of 69 mutations (59 SNPs and 10 indels) were detected by multiplex PCR, and 21 loci were significantly correlated with litter size in Nubian goat. The combined haplotype analysis revealed that AACC genotype in *KISS-1* gene and AAGG genotype in *KTLG* gene are notably lower than that of other combination genotypes in first born. This study suggests that these loci can be considered as effective SNP markers for genomic selection in Nubian goat breeding. However, further research with large sample sizes is needed to confirm the genetic effects of these mutations and molecular mechanisms.

## Supplement

10.5194/aab-64-375-2021-supplement Table S1 contains primers and PCR condition applied for pooled-DNA sequencing for the 43 candidate loci. Table S2 contains the information of 38 polymorphic loci (29 SNPs and 9 indels) of 23 genes identified by DNA pooling and the primers of multiplex PCR. Date S3 contains the information of 69 polymorphic loci (59 SNPs and 10 indels). Table S4 contains the annotation on the significant mutation loci linked with litter size in Nubian goat. The supplement related to this article is available online at: https://doi.org/10.5194/aab-64-375-2021-supplement.

## Data Availability

Data used and analyzed during this study are available from the corresponding author upon reasonable request.
