# Supplementary material for: Population validation of reproductive gene mutation loci and association with the litter size in Nubian goat
Source: Arch Anim Breed. 2021 Sep 17;64(2):375–86. doi: 10.5194/aab-64-375-2021 (PMC8461558; doi:10.5194/aab-64-375-2021)
Supplement: Table S1 contains primers and PCR condition applied for pooled-DNA sequencing for the 43 candidate loci. Table S2 contains the information of 38 polymorphic loci (29 SNPs and 9 indels) of 23 genes identified by DNA pooling and the primers of multiplex PCR. Date S3 contains the information of 69 poly [file aab-64-375-supplement.zip › Table S2.docx]

SUPPLEMENTARY MATERIALS

Population Validation of Reproductive Gene Mutation Loci and Association with the Litter Size in Nubian goat

Sanbao Zhang ^1^, Xiaotong Gao ^1^, Yuhang Jiang ^1^, Yujian Shen ^1^, Hongyue Xie ^1^, Peng Pan ^1^ , Yanna Huang ^1^, Yingming Wei ^2^ and Qinyang Jiang ^1^

**Table S2.** The information of 38 polymorphic loci (29 SNPs and 9 InDels) of 23 genes were identified by DNA pooling and the primers of multiplex PCR.

| **Gene** | **Sequencing results of pooled DNA/ Target loci** | **ID** | **Chromosome** | **Insert_start** | **Insert_end** | **F_primer** | **R_primer** |
| --- | --- | --- | --- | --- | --- | --- | --- |
| POU1F1 | 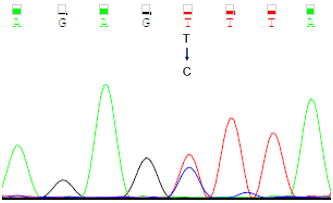 | g.34236170 | NC_030808.1 | 34236089 | 34236250 | TTTCTACTTTGGCTGGAGAAGAGAAGG | CCTGAGGATGGCTGAAGAACTAAAC |
| INHA | 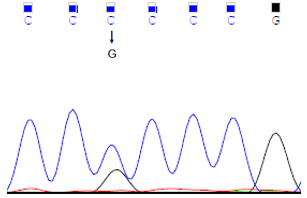 | g.28318345 | NC_030809.1 | 28318223 | 28318428 | CTGCCAGGTCTAAAAAGCGTTA | GCAGCTGCCAATCCCAAAAATA |
| CYM | 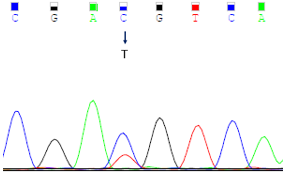 | g.87627082 | NC_030810.1 | 87627004 | 87627160 | CTGGCAGTCAGATCACACTTCC | GATGCATACCTGGCTGGTATAGG |
| NGF-1 | 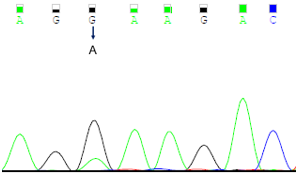 | g.92538023 | NC_030810.1 | 92537858 | 92538046 | CACACAAGCTGCTTCCAAAAAT | GGTTCATCCGGATCGACACA |
| INHβA | 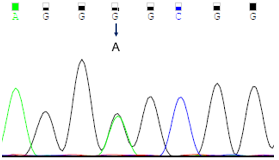 | g.40862949 | NC_030811.1 | 40862779 | 40862991 | GGAGCGATGATCCAGTCATTCC | CTCCTGGGCAAGAAGAAGAGGAA |
| KITLG | 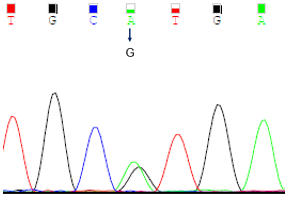 | g.18047585 | NC_030812.1 | 18047457 | 18047658 | GTATCATGGGATATTTGAGGGCCTAAA | GCTAGTCCAAGAATTTCTAATAGAGTTGGA |
|  | 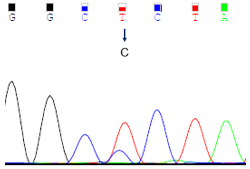 | g.18048381 | NC_030812.1 | 18048267 | 18048492 | CTTGGACTAGCAAAGTCAAATAAAAGGTA | AGTGAATTTCCCTTGCCAATTTTCTTTT |
|  | 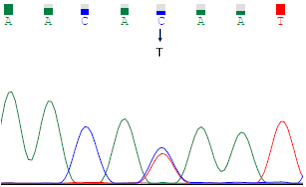 | g. 18048487 | NC_030812.1 | 18048467 | 18048695 | ATCAGTGTCCACAGCTTCTTCAT | TCAGTAGCATCATCCACTGTTCATTT |
|  | 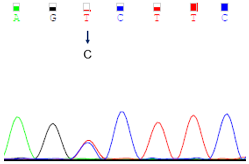 | g.18047236 | NC_030812.1 | 18047227 | 18047409 | CTATGAGGCAGACCTGAACCTAC | ACGTGTCCCAGATTGCACATAC |
|  | 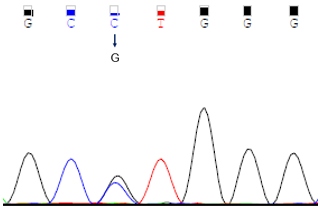 | g.18152019 | NC_030812.1 | 18151850 | 18152074 | TGCAGATAGTCCACGCATTGAG | GCTTCGCTTGATCTGTCCAACT |
| IGF1 | 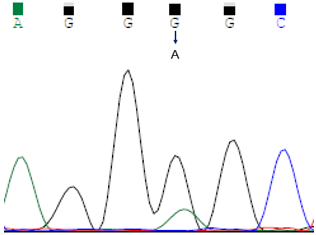 | g.64943047 | NC_030812.1 | 64943004 | 64943211 | TCTCCCTCTTCTGGCAAAGTTA | CCACGAATATTCCTTTCATACGGGTAA |
|  | 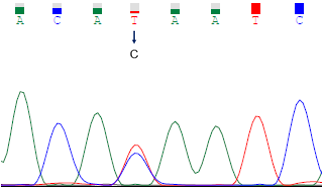 | g.64943050 | NC_030812.1 | 64942821 | 64943014 | GGCATGAAGACACACACATCTG | CCAGGTTCTAGGAAATGAGATCATTCC |
| MARCH1 | 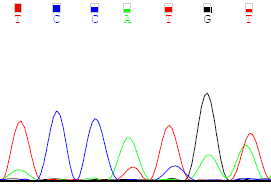 | g.1885629-1885646 | NC_030813.1 | 1858635 | 1858811 | TGATGACTAACCAAGATACTGTGCAT | TGGCAATCTCATATGAGTGAATCATTCATT |
|  | 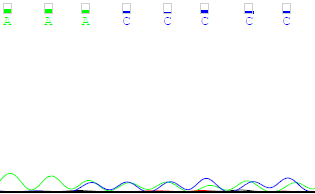 | g.1885505-1885511 | NC_030813.1 | 1885491 | 1885719 | GAAAATGAGGCCAAGTTCCTGAG | CCTTAGAACAGGAGGGCTAGACA |
|  | 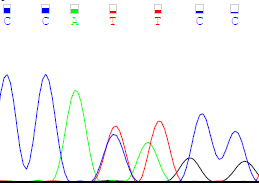 | g.1947289-1947293 | NC_030813.1 | 1947207 | 1947333 | GCTGTCCTGAAAATAACTTCATCAATACTA | CATTGACAGACGAATGGTCCATATATACAA |
|  | 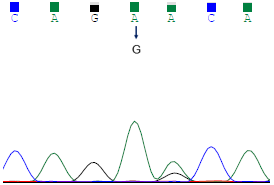 | g.1947289 | NC_030813.1 | 1947069 | 1947219 | AAAACTGCAGTCGTTTTTATGGCTG | CTCCTAGGCAACACCACGAGGAAA |
| GnRHR | 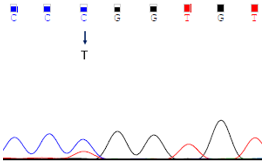 | g.84161773 | NC_030813.1 | 84161663 | 84161880 | CATCTTCTCTTTCCTCTGAGTCCAAT | CAATTACAATAAACATCAGAAGTGCCAGAA |
| CSN1S1 | 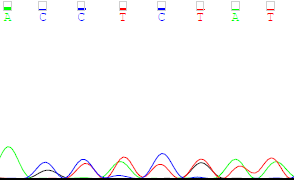 | g.859864555-85986461 | NC_030813.1 | 85986385 | 85986599 | TAATTCTAAAAGTCTCAGAGGCAGTAACAA | TGCCCAGAAAGAAATCTTTATTTTGTCTCT |
| PDGFRB | 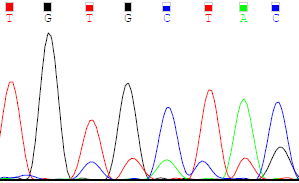 | g.48948299-48948308 | NC_030814.1 | 48948217 | 48948433 | AGAAGTGCTGGGAAGAGAAGTTC | TGCTAAAATAAACTACACTCACATCCCTT |
| GDF9 | 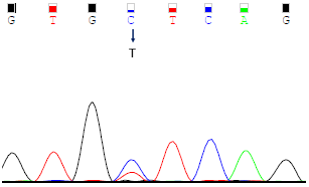 | g.66027701 | NC_030814.1 | 66027576 | 66027799 | GGCCTCCCACAAGAGGAATATT | GATCTTACACCCTCAGCAGCTT |
| GnRH1 | 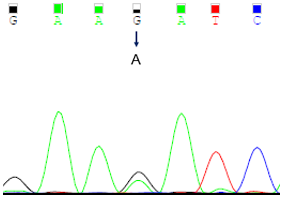 | g.72491357 | NC_030815.1 | 72491227 | 72491450 | TACAGACGTTAAATGGGTCACAGATTTC | GCATGATCCAACTCTCTAGCACT |
| NEDD4 | 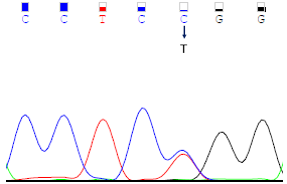 | g.48710049 | NC_030817.1 | 48709960 | 48710169 | TTCCCGAGACTTAAGGTTTTCCTTTA | CTGTCTACCGACAGAGATCTTTGT |
|  | 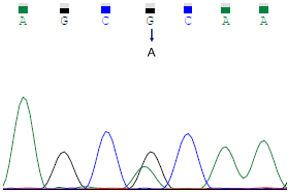 | g.48709794 | NC_030817.1 | 48709678 | 48709902 | CTGCACTGCAGCAAAAGAAAAAA | AAACAAGGTTGGCAGTGTTTGAG |
| CDH26 | 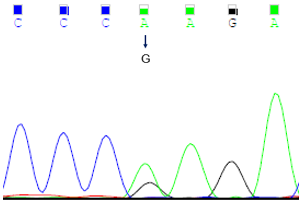 | g.55881105 | NC_030820.1 | 55881008 | 55881222 | TTTAAAATCCCTTCATTGGTCTCAGGATC | CCAAGTTGTTGTCTCTCTATGTTTTCTTTC |
| FOLR1 | 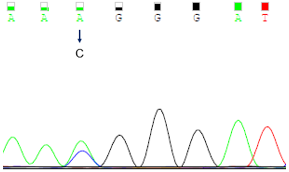 | g.31625869 | NC_030822.1 | 31626717 | 31626943 | GGTGAGCACTCATAGAGACAGAT | AGGTGGGAACATTCTGGTTGTG |
|  | 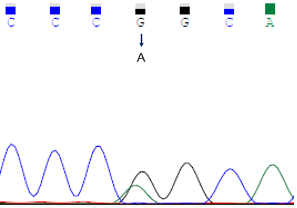 | g.31626833 | NC_030822.1 | 31625775 | 31625969 | GCACACAATATGTGTAAAAGAGTCGA | GATCACACCACTACTGTGCCTT |
| PGR | 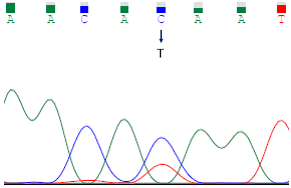 | g.74589762 | NC_030822.1 | 74589577 | 74589779 | CTCTGGGTCCAGCGTCTTC | GGCGTCATGACTGAGCTGA |
| KISS-1 | 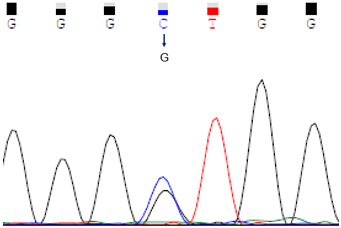 | g.1341674 | NC_030823.1 | 1341511 | 1341705 | GTCTCATCCAGGGTGAGTGATA | GCTCTTTCTGGGTAAGGGAGGAT |
| ATBF-1 | 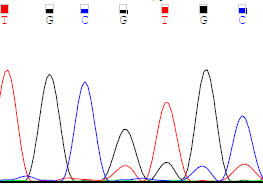 | g.39535922-39535933 | NC_030825.1 | 39535933 | 39536123 | GTTTTGACTGGAGTCATTGTTATTG | AGCTTGGTGGGAATTTTCAC |
|  | 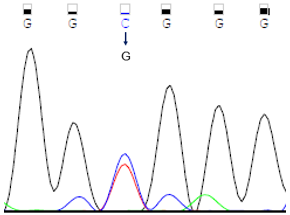 | g.39535717 | NC_030825.1 | 39535691 | 39535940 | AAGTGGCCAAAGTATTTTAGCATCAGT | GGCAGCTTTGAACCTCCCATA |
|  | 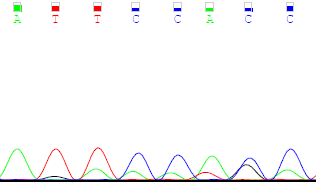 | g.39575627-39575632 | NC_030825.1 | 39575463 | 39575691 | GCTTTGGCTGAGAACACAGATA | GTGCTTTGCATCCATCGTGA |
| GHR | 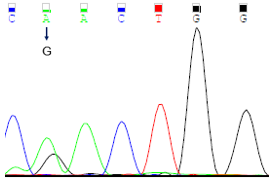 | g.32134266 | NC_030827.1 | 32134071 | 32134299 | CCAAACTCAGAACTCAAGCACAG | GTCTTAGAGCCTCAAGACACCTT |
| PRLR | 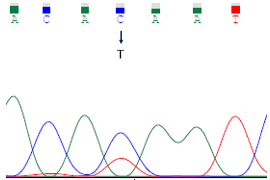 | g.39056974 | NC_030827.1 | 39056964 | 39057189 | TTGCAAACACTCAGAATAAAGTGGTG | GGACTGGGATGGATTCTCTCCT |
|  | 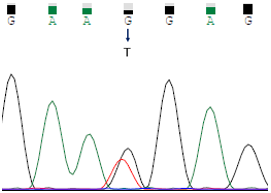 | g.39081154 | NC_030827.1 | 39081144 | 39081367 | AAAACCTGACTCTGTTAGAAGAATCAGC | GGTAGTGTCATTCTAATCCTGTTCTTGAG |
|  | 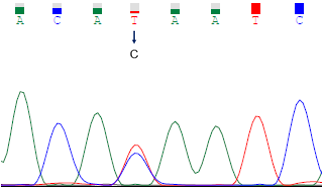 | g.32134266 | NC_030827.1 | 39081357 | 39081490 | AGAGTACGTGGAATGCACTCTA | TGAAGGCCTTTTCCAGAGAATATACCTA |
|  | 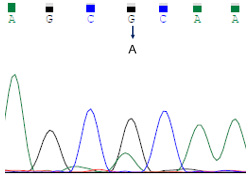 | g.39081047 | NC_030827.1 | 39080937 | 39081154 | TAAAAGCACATTGCCTAGTATTCTTGAGAG | GCAAAGGTTAAGCAACTGGTCTT |
| CTNNB1 | 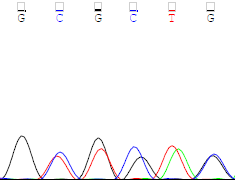 | g.1371229-13712314 | NC_030829.1 | 13712137 | 13712360 | GCAGTAGGTGGTAAAACTTTGCTA | TGGATCCACTAATTGAGTGTCACTTTG |
| KDM6A | 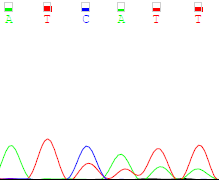 | g.27180767-27180771 | NW_017189516.1 | 27180613 | 27180821 | AGTTGTTAATTGATTAGCTTCTGAGTAGAAA | AAAAAGCCTAAATGCATGAAAAGTTGA |
