# Supplementary material for: Population validation of reproductive gene mutation loci and association with the litter size in Nubian goat
Source: Arch Anim Breed. 2021 Sep 17;64(2):375–86. doi: 10.5194/aab-64-375-2021 (PMC8461558; doi:10.5194/aab-64-375-2021)
Supplement: Table S1 contains primers and PCR condition applied for pooled-DNA sequencing for the 43 candidate loci. Table S2 contains the information of 38 polymorphic loci (29 SNPs and 9 indels) of 23 genes identified by DNA pooling and the primers of multiplex PCR. Date S3 contains the information of 69 poly [file aab-64-375-supplement.zip › Table S1.docx]

SUPPLEMENTARY MATERIALS

Population Validation of Reproductive Gene Mutation Loci and Association with the Litter Size in Nubian goat

Sanbao Zhang ^1^, Xiaotong Gao ^1^, Yuhang Jiang ^1^ , Yujian Shen ^1^, Hongyue Xie ^1^, Peng Pan ^1^, Yanna Huang ^1^, Yingming Wei ^2^ and Qinyang Jiang ^1^

**Table S1.** Information of primers used to verification SNPs in Nubian goat.

| **Gene** | **Forward primer (5'→3' on plus strand）** | **Reverse primer (5'→3' on minus strand)** | **Product length(bp)** | **Function** |
| --- | --- | --- | --- | --- |
| *MARCHF1* | ACTGTGCATCAAGAATGTGGAAA | TGAGGTACATGGAAATGGCAAA | 185/192 | Indel detection |
|  | GTTCATTGTGTGTTTCTAATGGCT | TGGTTTGGAGCACAATCAAGTAA | 156/174 |  |
|  | ACTTCACTGTGCATAATAAGCTCTA | CACCACGAGGAAACGAAA | 217/232 |  |
| *KDM6A* | AGAGTTCATTCACAGATTCCACTT | AAAAGAATCCAGGTGGGTGTCA | 240/246 | Indel detection |
|  | AATTTTGACACCCACCTGGA | CACTGAGCATGCAAAGGAATACA | 141/146 |  |
| *CSN1S1* | GCTGGAAGCAGTTCGTCA | GGGTTGATAGCCTTGTATGTT | 159/170 | Indel detection |
| *CTNNB1* | AAATCGAGGGAGCACCTGAA | GTGGCCTCTTGTCAGCACTAA | 162/188 | Indel detection |
| *GDF9* | ACCCATCTAGTGTTCTCGCC | GCCATCTAAAGCGCAGAGTT | 177/189 | Indel detection |
|  | GCAACCTGGTGATAAAAGAG | CAAAACACTCAAAGGGCTAT | 761 | SNP detection |
|  | ATCCCACCCTGACGTTTAAGGC | TCCTCCCAAAGGCATAGACAGG | 1170 |  |
| *GHR* | TCCTAGAACCCTGATTCCC | CTCCATTTTCCAGTTGTGC | 161/175 | Indel detection |
| *PDGFRB* | CCAGCTCAGGATGGGTCT | CCAGCATGGGCACATAGTC | 296 | Indel /SNPdetection |
|  | ACCTGAATCTGTCTGGTGTGT | CAGAAAGGGAAAGGGACATGC | 222/227 |  |
|  | GCTGGGTGAGGGCTACAAAA | AAACACCAGTGCGTCACAGT | 120 |  |
| *ATBF1* | CTCTGTCACCTCCTTCTGC | AGCTTTGAACCTCCCATAA | 313/301 | Indel detection |
|  | CCCCTTAATTCCAGTGAGGTTT | GCATCCATCGTGAGCGTGTA | 166/172 |  |
| *CYM* | GAGAAAGGAGGAGAGCTGGG | CACCCATGGCCCTTCTAAGA | 195 | SNP detection |
| *CDH26* | ATGGGACTACATAAACCTA | CCAGTCACAGGGACGAGAT | 680 | SNP detection |
| *KISS1* | CCCGCTGTAACTAGAGAAAG | CATCCAGGGTGAGTGATACT | 377 | SNP detection |
| *GnRHR* | TTTGCTTTAGCACCCTGTTG | GAGCCCAAGCTCTCAGAGATAA | 849 | SNP detection |
| *GNRH1* | ACCTCTGTCCTCACACCCTA | CATTTATGCCATTTTATTCC | 430 | SNP detection |
| *POU1F1* | CGATCATCTCCCTTCTT | AATGTACAATATGCCTTCTGAG | 450 | SNP detection |
| *NEDD4* | GTTTCGTTCCTACCAACTTCCT | TGCTGCTATCCAAATCATCTCC | 633 | SNP detection |
| *KITLG* | GAAACTACCAGCCGTCGCCCAA | CGCGCCCTACTTACTTGTGTCT | 587 | SNP detection |
|  | AAACTGCCCTTTACAATGCC | ATCTGGAATAACACTGCTTG | 323 | SNP detection |
|  | GCCTCCAGAAGCATCTAAC | CAAGTTCAGACCCACATCC | 275 |  |
|  | TAGCATCATCCACTGTTCAT | ATGTCCTCCTCGGCAGAT | 298 |  |
|  | GACTTAGGAGGAATCACTGA | AACGCAATGTCCATCTTG | 386 |  |
|  | TGAGTGAATTTGGCAGGAA | GGTGGTAGGATATGGCAATA | 250 |  |
|  | CCTTCAATAGAGTGACACAG | CAGGTCCTTACAGCCATTA | 346 |  |
| *FOLR1* | CCAGGAAGATATTGTCTCAA | GCTCTAGGCATTGTTCTGA | 499 | SNP detection |
|  | GTCCCTCCACCTGATGTT | CCTCCTCAGACCAGAATT | 414 | SNP detection |
| *NGF* | CTGGGAGAGGTGAACATC | ACAGGTTGAGGTAGGGAG | 319 | SNP detection |
| *INHA* | AGCTGCCAATCCCAAAAATA | TGACACCAGATGGGACAGGA | 284 | SNP detection |
| *INHβA* | ATACGGATTGCCTGTG | CTCACAGTAGTTGGCGT | 333 | SNP detection |
| *PRLR* | AGTGAGAGTTATGGAAGGATG | AAGGTTAAGCAACTGGTCTT | 443 | SNP detection |
|  | GCTATTTGCACAAGAGGAGG | AATGAGGATGGAAGTCAGAG | 200 |  |
|  | ACATTCAGCAAGGAGCAAGA | TCCTCCTCTTGTGCAAATA | 280 |  |
| *IGF1* | GGGTATTGCTAGCCAGCTGGT | CCGGGCATGAAGACACACACAT | 601 | SNP detection |
| *SIRT3* | CCTTCAGACCCCTCTTCCTG | CCATTAGGCCTGCTGACAAA | 213 | SNP detection |
| *PGR* | ACTGAGCTGAAGGCGAAGGGTC | GGTGTCGCCAGCCTTGCTCTCCG | 513 | SNP detection |
