# Supplementary material for: Population validation of reproductive gene mutation loci and association with the litter size in Nubian goat
Source: Arch Anim Breed. 2021 Sep 17;64(2):375–86. doi: 10.5194/aab-64-375-2021 (PMC8461558; doi:10.5194/aab-64-375-2021)
Supplement: Table S1 contains primers and PCR condition applied for pooled-DNA sequencing for the 43 candidate loci. Table S2 contains the information of 38 polymorphic loci (29 SNPs and 9 indels) of 23 genes identified by DNA pooling and the primers of multiplex PCR. Date S3 contains the information of 69 poly [file aab-64-375-supplement.zip › Table S4.docx]

SUPPLEMENTARY MATERIALS

Population Validation of Reproductive Gene Mutation Loci and Association with the Litter Size in Nubian goat

Sanbao Zhang ^1^, Xiaotong Gao ^1^, Yuhang Jiang ^1^, Yujian Shen ^1^, Hongyue Xie ^1^, Peng Pan ^1^, Yanna Huang ^1^, Yingming Wei ^2^ and Qinyang Jiang ^1,^ *

**Table S3.** Annotation on the significant mutation loci linked with litter size in Nubian goat.

| Gene | Chromosome | Start | End | Ref | Alt | No. of rs | EFFECT | HGVS_C | HGVS_P |
| --- | --- | --- | --- | --- | --- | --- | --- | --- | --- |
| *POU1F1* | NC_030808.1 | 34236170 | 34236170 | A | G | rs641347841 | synonymous_variant | c.837T>C | p.Ser279Ser |
| *INHA* | NC_030809.1 | 28318305 | 28318305 | G | ins_C |  | 5_prime_UTR_variant | c.-542_-541insC, |  |
|  |  | 28318345 | 28318345 | C | G |  | 5_prime_UTR_variant | c.-502C>G |  |
|  |  | 28318349 | 28318349 | G | C |  | 5_prime_UTR_variant | c.-498G>C |  |
| *KITLG* | NC_030812.1 | 18047318 | 18047318 | G | A | rs658181751 | 3_prime_UTR_variant | c.*1707C>T |  |
|  |  | 18048657 | 18048657 | G | T | rs671224715 | 3_prime_UTR_variant | c.*368C>A |  |
|  |  | 18152042 | 18152042 | G | A |  | intergenic_region | n.18152042G>A |  |
| *MARCHF1* | NC_030813.1 | 1858739 | 1858739 | G | A |  | intergenic_region | n.1858739G>A |  |
|  |  | 1885620 | 1885620 | A | G |  | intron_variant | c.111+25480A>G |  |
| *GDF9* | NC_030814.1 | 66027701 | 66027701 | C | T | rs662668357 | missense_variant | c.818C>T | p.Ala273Val |
| *NEDD4* | NC_030817.1 | 48709794 | 48709794 | G | A | rs659476749 | intron_variant, | c.318+8909C>T, |  |
|  |  | 48710049 | 48710049 | G | A | rs639719147 | synonymous_variant | c.187C>T, | p.Ser23Ser |
| *PGR* | NC_030822.1 | 74589762 | 74589762 | C | T |  | synonymous_variant | c.31G>A | p.His48His |
| *KISS1* | NC_030823.1 | 1341600 | 1341600 | A | G | rs662420130 | missense_variant | c.104-1006T>C | p.Ala11Thr |
|  |  | 1341674 | 1341674 | C | G | rs645841275 | intron_variant | c.104-1080G>C |  |
| *ATBF1* | NC_030825.1 | 39535967 | 39535967 | C | T |  | intron_variant | c.3478+7382C>T |  |
|  |  | 39535996 | 39535996 | C | T |  | intron_variant | c.3478+7411C>T |  |
| *GHR* | NC_030827.1 | 32134187 | 32134187 | G | A |  | intron_variant | c.-12+40511C>T |  |
|  |  | 32134266 | 32134266 | T | C |  | intron_variant | c.-12+40432A>G |  |
| *CTNNB1* | NC_030829.1 | 13712297 | 13712314 | TACTTGGCTGTGCACAGT | del_TACTTGGCTGTGCACAGT | rs646881456 | intron_variant | c.1684-238_1684-222delACTTGGCTGTGCACAGT |  |

**Note:** HGVS: Human Genome Variation Society; Ser: Serine; Ala: alanine; Val: valine; His: histidine; Thr: threonine.
