# Supplementary material for: Population validation of reproductive gene mutation loci and association with the litter size in Nubian goat
Source: Arch Anim Breed. 2021 Sep 17;64(2):375–86. doi: 10.5194/aab-64-375-2021 (PMC8461558; doi:10.5194/aab-64-375-2021)
Supplement: Table S1 contains primers and PCR condition applied for pooled-DNA sequencing for the 43 candidate loci. Table S2 contains the information of 38 polymorphic loci (29 SNPs and 9 indels) of 23 genes identified by DNA pooling and the primers of multiplex PCR. Date S3 contains the information of 69 poly [file aab-64-375-supplement.zip › aab-64-375-2021-supplement-title-page.pdf]

Supplement of Arch. Anim. Breed., 64, 375–386, 2021
https://doi.org/10.5194/aab-64-375-2021-supplement
© Author(s) 2021. CC BY 4.0 License.

Open Access
Archives Animal Breeding

Supplement of

Population validation of reproductive gene mutation loci and association with the litter size in Nubian goat

Sanbao Zhang et al.

Correspondence to: Qinyang Jiang (jiangqinyang2013@gxu.edu.cn)

- aab-64-375-2021-supplement-title-page.pdf
- Date S3.xlsx
- Table S1.docx
- Table S2.docx
- Table S4.docx

The copyright of individual parts of the supplement might differ from the article licence.
